# Assessment of mortality from COVID-19 in a multicultural multi-ethnic patient population

**DOI:** 10.1186/s12879-021-06762-9

**Published:** 2021-10-29

**Authors:** Satish Chandrasekhar Nair, Huda Imam Gasmelseed, Asad Afroz Khan, Ibrahim Nageh Khafagy, Jayadevan Sreedharan, Aqeel Aziz Saleem, Hashim Ibrahim Abdrhman, Ahmed Husain Alhosani, Amatur Rahman Siddiqua, Amna Riaz Ahmed, Aya Imad Shubbar, Abdul Rahman Aleissaee, Abdulrahman Wael Alanqar, Alan Mohammad Hamadeh, Fatmah Ali Safdani, Fuad Wardan Habbal, Haneen Bassam Choker, Khlood Mustafa Bashir, Maitha Ali Alblooshi, Majd Munir Farajallah, Mohamed Nasir Alzaabi, Rajish Sanjit Shil, Saif Saeed Alshehhi, Wafa Fayez Douleh

**Affiliations:** 1 Department of Academic Affairs, College of Medicine & Health Sciences, Tawam Hospital, UAE University, Academic Affairs, Post Box 15258, Al Ain, UAE; 2 Department of Infectious Diseases, Internal Medicine, Al Ain Hospital, Al Ain, UAE; 3 Department of Infectious Diseases, Internal Medicine, Tawam Hospital, Al Ain, UAE; 4 Department of Pharmacy, Al Ain Hospital, Al Ain, UAE; 5 Department of Community Medicine, Gulf Medical University, Ajman, UAE; 6 Department of Academic Affairs, Internal Medicine, Tawam Hospital, Al Ain, UAE; 7 Department of Infectious Disease, Internal Medicine, Al Ain Hospital, Al Ain, UAE

**Keywords:** Gulf, United Arab Emirates, Infectious disease, Comorbidity, Kidney disease

## Abstract

**Background:**

Studies indicate that ethnicity and socioeconomic disparity are significant facilitators for COVID-19 mortality. The United Arab Emirates, distinctly has a population of almost 12% citizens and the rest, immigrants, are mainly unskilled labourers. The disparate socio-economic structure, crowded housing conditions, and multi-ethnic population offer a unique set of challenges in COVID-19 management.

**Methods:**

Patient characteristics, comorbidities, and clinical outcomes data from the electronic patient medical records were retrospectively extracted from the hospital information system of the two designated public COVID-19 referral hospitals. Chi-square test, logistic regression, and odds ratio were used to analyse the variables.

**Results:**

From, the total of 3072 patients, less than one-fifth were females; the Asian population (71.2%);followed by Middle Eastern Arabs (23.3%) were the most infected by the virus. Diabetes Mellitus (26.8%), hypertension (25.7%) and heart disease (9.6%) were the most prevalent comorbidities observed among COVID-19 patients. Kidney disease as comorbidity significantly diminished the survival rates (Crude OR 9.6, 95% CI (5.6–16.6), p < 0.001) and (Adjusted OR 5.7 95% CI (3.0 – 10.8), p < 0.001), as compared to those patients without kidney disease. Similarly, the higher age of patients between 51 and 65 years, significantly decreased the odds for survival (Crude OR 14.1 95% CI (3.4–58.4), p < 0.001) and (Adjusted OR 12.3 95% CI (2.9 – 52.4), p < 0.001). Patient age beyond 66 years, further significantly decreased the odds for survival (Crude OR 36.1 95% CI (8.5–154.1), p < 0.001), and (Adjusted OR 26.6 95% CI (5.7 – 123.8), p < 0.001).

**Conclusion:**

Our study indicates that older ages above 51 years and kidney disease increased mortality significantly in COVID-19 patients. Ethnicity was not significantly associated with mortality in the UAE population. Our findings are important in the management of the COVID-19 disease in the region with similar economic, social, cultural, and ethnic backgrounds.

## Background

The COVID-19 viral infection outbreak, following the first report from Wuhan, China, in December 2019, has been headlined for global attention [1]. The rapid infectivity of the severe acute respiratory syndrome virus strain (SARS-CoV-2), a novel and highly communicable pathogen, prompted the World Health Organization (WHO) to escalate the outbreak to a pandemic on March 11, 2020 [2]. The SARS-CoV-2 virus strain causes severe acute respiratory failure, needing hospitalization for less than thirty percent of those severely ill, eroded healthcare resources and medical supplies, culminating in a global public health crisis that mankind had not witnessed before [3]. Since the first report of the outbreak, the virus has infected almost 109 million people and caused almost 2.4 million fatalities in 218 countries of the world up until February 14, 2021 [4].

The United Arab Emirates (UAE) occupies a strategic location between Europe, Asia, and Africa, and is geographically closer to China. The UAE is a member country of the Gulf Cooperation Council, a political and economic union of five other Arab states, including Bahrain, Kuwait, Oman, Qatar, and Saudi Arabia [5]. In recent years, the UAE has transformed from a traditional to a state-of-the-art modern country, diversifying away from financial reliance on oil, to develop into a knowledge-based economy [6, 7]. The strategic location and a growing economy have catapulted the UAE to become the epicenter for travel, transit, trade, and business [7].

Distinctively, the citizens of the UAE contribute to less than twelve percent of the total nine million population. The immigrants (expatriates) from almost eighty other countries contribute to the bulk of the population. More than two-thirds of the expatriate population are unskilled laborers, especially from South Asia, and Africa [6] [8]. The ability to tolerate relatively poor working and living conditions, and to work long hours has enhanced the contribution of the unskilled laborers to approximately ninety-eight percent of the overall labor force in the private sector [8]. The UAE was exposed to other coronavirus epidemics, such as the Severe Acute Respiratory Syndrome (SARS) outbreak in 2002 and the spread of Middle East Respiratory Syndrome (MERS) in 2012 [9, 10]. In spite of this, limited information about patient characteristics and clinical management of the epidemics are available [9]. The UAE is the second-worst affected country, among the GCC countries, by the COVID-19 disease, preceded by Saudi Arabia with a total of 361,000 positive cases and approximately 6000 deaths [11]**.** The COVID-19 outbreak was first reported in the United Arab Emirates on February 29, 2020, when the first five cases were identified. Almost ten weeks later, the number of positive COVID-19 cases surged to 1695. Despite, 346,000 positive COVID-19 cases, the number of deaths in the UAE remained low at less than 0.3% until February 14, 2021 [4, 12]. Several reports indicate that ethnicity and socioeconomic disparity are significant contributors to the spread of infectious diseases [13, 14]. The disparate socioeconomic structure among the immigrants (expatriates), and the multiethnic multicultural patient population in the UAE, offers a unique set of challenges in the clinical outcomes of COVID-19 patients [15]. Therefore, the objectives of this study were to assess the mortality rate for COVID-19 infected patients in a multicultural multiethnic population of the United Arab Emirates and to describe patient characteristics and the risk factors.

## Methods

### Study design

The design comprised a retrospective observational study of patients who tested positive for SARS-CoV-2 virus strain [16]. The retrospective nature of the study restricted the involvement of patients or the public in the design.

### Setting

Data was collected from COVID-19 patients hospitalized at the only two public hospitals in Al Ain: these hospitals catered to more than 98% of the total positive COVID-19 caseload. Largely, the two public hospitals address the secondary and tertiary care needs of the population of the eastern region of the United Arab Emirates. The study period was between March 1 and June 30, 2020, during the peak of the contagion in the UAE.

### Participants

All the patients hospitalized for COVID-19 disease at the two public hospitals were selected for the study provided they met the inclusion criteria: a) hospitalized patients 18 years of age or older, b) all nationalities, c) had a confirmed positive COVID-19 RTPCR test result, d) both male and female, and e) were seen at the emergency department, in-patient units, and the designated COVID-19 screening tents. Patients below the age of 18 years (pediatric), and with incomplete (demographic information) or a missing result (Rapid Antigen Test instead of the RT-PCR for SARS-CoV-2 virus) documentation were excluded from the study.

### Variables

Demographic information collected from the patient electronic medical records included age, gender, ethnicity, and smoking status. Ethnicity was categorized into Middle Eastern Arabs (including UAE nationals), Asians, and Others (America’s, Europe, and Africa). Age was categorized into 18–30, 31–50, 51–65, and > 66 years. The smoking status was categorized as the patients who smoked cigarettes (Smoking), and those who abstained from smoking of any kind (Non-Smoking). The majority (98.6%) of the patients with smoking habits were cigarette smokers; less than 1.4% were water-pipe (Sheesha) smokers. Socioeconomic status was derived from the type of housing the patients lived in. Single shared housing reflected lower economic status, provided for the unskilled expatriates with low income. UAE nationals and skilled expatriates were accommodated in a family housing setup. If the socioeconomic status was not clear, then the data was recorded as undisclosed/unknown. Comorbidities such as kidney, diabetes mellitus, heart, hypertension, respiratory, hematological, immunocompromise, liver, diseases, and obesity were recorded from the patient medical records. Chronic kidney disease was prevalent in the majority of the patients with kidney dysfunction. The Body Mass Index of ≥ 30 was tabulated as the “obesity risk.” Approximately, less than two-thirds (408/936) of the patients had controlled diabetes, hypertension, and heart disease.

### Data source

Laboratory data was collected for the patients positive for the SARS-Cov-2 virus, confirmed using the Reverse Transcriptase Chain Reaction (RTPCR), from the Laboratory Information System and the Radiological data from the PACS (Picture Archiving and Communication System) of the Hospital Information System (Cerner, USA). Furthermore, the demographics, comorbidities, clinical progress, and patient outcomes data were abstracted from the patient electronic medical records of the same hospital information system. Cerner system has been validated and used by all of the Abu Dhabi Health Services Company-managed public hospitals in the UAE since 2008 [17]. The data collected was recorded in the Microsoft Excel sheets, secured for authorized access by using a password. Standard statistical tests were applied to estimate the difference between the various variables, and are described under the ‘statistical analysis’ section.

### Bias

The COVID-19 Preparedness at Tawam and Al Ain hospitals (COPTA), a research group consisting of twenty-four physician-researchers, extracted data for the study, but were blinded to the study hypothesis. Interrater reliability, Cohen’s kappa coefficient, was used to assess the differences in the data between the—abstractors [18]. Selection and operator bias were excluded by involving multiple data abstracters.

### Study size

The study period was during the peak of the contagion in the UAE, therefore all the hospitalized COVID-19 patients, at both the public hospitals were selected for the study. From a total of 3452 patients, 3072 patients that met the inclusion criteria were shortlisted and their data abstracted. The remainder of 380 patients were excluded from the study for the reasons listed under the ‘participants’.

### Statistical methods

Data was analyzed using SPSS (IBM, Chicago) Version 26. All hospitalized patients during the study period with COVID-19 were selected for the study, and shortlisted based on the inclusion criteria. The interrater reliability test was applied to assess the agreement of the data collected between the data abstractors. The Cohen Kappa for the 24 data abstractors was 0.79, indicating good agreement. Descriptive and inferential statistical methods were used to analyze the data. First, the frequency and percentages were taken for all variables, and then the important variables were cross-tabulated with the outcome (Deceased/Survived). The Chi-Square test was used to find the association between the outcome variable and other independent variables. For 2 × 2 tables, the Fisher’s exact test was used wherever the expected cell value was less than 5, and if more than 20% of cells have expected frequency less than 5, The level of significance was taken as p < 0.05. The simple logistic regression was used to determine the crude Odds Ratio (OR), and multiple logistic regression, to determine the adjusted Odds Ratio. The enter method was used to calculate the Odds Ratio. The variables were adjusted to each other and a. 95% confidence interval was determined to find the statistically significant variables.

### Ethics statement

The methods of the study were carried out per the International Conference for Harmonization (ICH), and Good Clinical Practice guidelines. Waiver of written informed consents for participation in this retrospective observational study was granted by the Abu Dhabi Technical and Scientific Human Research Ethics Committee, a central research ethics committee at the department of health Abu Dhabi, UAE.

## Results

Although there were three thousand four-hundred and fifty patients presented to the public hospitals during the study period, only three thousand and seventy-two, meeting the inclusion criteria constituted the study population, The remainder three hundred eighty and patients were excluded given the anomalies, in their COVID-19 test result and, or the age recorded (pediatric age). More than half (1630/3072, 53%) of the patients were in the age group between 31 and 50 years (Table 1), followed by older patients above 51 years of age (966/3072, 31.5%). Less than one-fifth of the total COVID-19 patients were females (659/3072, 21.5%), the majority being male patients. COVID-19 was widespread among the Asian population (2187/3072, 71.2%), followed by Middle Eastern Arabs (716/3072, 23.3%). There was a higher likelihood of patients with a smoking habit getting infected by the virus. More than 80% of the smokers contracted COVID-19 disease, as compared to those who did not smoke (Table 1). Housing conditions reflect the socio-economic structure in the United Arab Emirates. The workers with low income live in single shared housing. The vast majority of the Asian population (691/734, 94.1%, p < 0.001), lived in single shared accommodation, compared to the Middle East Arab population (23/734, 3.1%), and others (20/734, 2.7%) (Table 1). In contrast, the majority of the Middle Eastern Arabs lived in Family accommodation (526/902, 58.3%) (Table 1).

**Table 1:** Patient characteristics and clinical outcomes of adult COVID-19 patients admitted to the public hospitals in AlAin, the eastern region of the UAE

Characteristics	Category	Numbers		Outcome				P
				Survived		Deceased		
		n	%	n	%	n	%	
Age (years)	18–30	476	15.5	462	99.6	2	0.4	< 0.001
	31–50	1630	53.1	1566	98.4	25	1.6	
	51–65	770	25.1	706	94.3	43	5.7	
	≥ 66	196	6.4	160	86.5	25	13.5	
Gender	Male	2413	78.5	2273	96.7	77	3.3	Not significant
	Female	659	21.5	621	97.2	18	2.8	
Nationality	Asian	2187	71.2	2080	97.4	55	2.6	< 0.005
	Middle East Arab	716	23.3	665	95.7	30	4.3	
	Others	167	5.4	149	93.7	10	6.3	
Smoking Status	Smoking	1712	85.8	1638	97.4	44	2.6	Not significant
	Non-Smoking	283	14.2	249	97.6	6	2.4	
Socioeconomic Status (Housing)	Family	877	55.3	843	96.1	34	3.9	< 0.005
	Single	710	44.7	699	98.5	11	1.5	
Socioeconomic Status (Single/Shared Housing)	Asian	669	–	659	98.5	10	1.5	**
	Middle East Arab	23	–	22	95.7	1	4.3	
	Others	18	–	18	100.0	–	–	
Socioeconomic Status (Family Housing)	Asian	300	–	292	97.3	8	2.7	Not significant
	Middle East Arab	510	–	488	95.7	22	4.3	
	Others	67	–	63	94.0	4	6.0	

**Expected cell values not sufficient to calculate the Chi-square statistic

Subgroup analysis of the data indicated a statistically significant (p < 0.001) positive association between age and survival. Higher age increased mortality rate. The mortality rate was less than 0.5% for the COVID-19 patients in the age group of 18–30 Years. Between 31–50 years, the death rate was less than 2% and increased to 5.7% and 13.5%, for the older age group of 51–65 years and 66 + years, respectively (Table 1). A statistically significant association between mortality and gender and whether the patients were smokers, or not was not observed (Table 1). A statistically positive association was observed between mortality and the nationalities. The Middle Eastern Arabs (4.3%) and other nationalities ((immigrants from America’s, Europe, and Africa) (6.3%), were more likely to succumb to COVID-19 infection, as compared to the Asian population (2.6%, p < 0.005) (Table 1). This is despite the large number of Asians who contracted COVID-19 infection (2080/3072, 97.4%). Almost three-fold higher mortality among the COVID-19 patients living in family accommodation (3.9%) was observed when compared with the patients living in single shared accommodation (1.5%, p < 0.05) (Table 1).

**Fig. 1.**
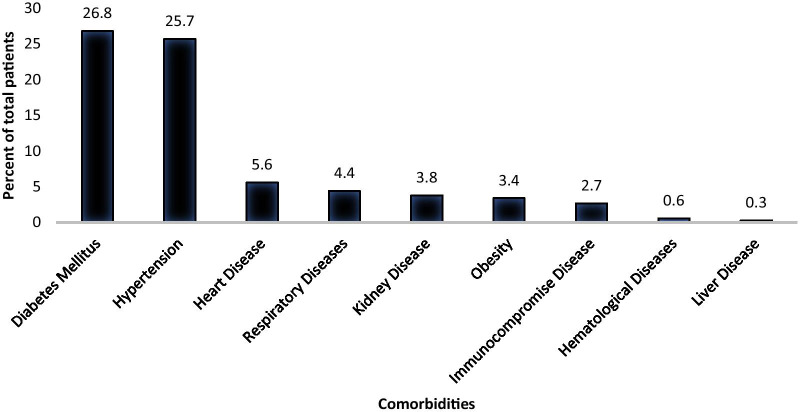
Percentage of total patients infected with COVID-19 presenting with various comorbidities

Diabetes Mellitus was the most predominant comorbidity observed among adult COVID-19 patients, more than one-quarter of the patients presented with Diabetes Mellitus (792/3072, 26.8%) (Fig. 1). Hypertension (759/3072, 25.7%) was the second most common comorbidity, followed by heart disease (162/3072, 5.3%). Other comorbidities such as respiratory (130/3072, 4.4%), kidney (112/3072, 3.8%), obesity (102/3072, 3.4%), immunocompromised (80/3072, 2.7%), diseases were below five percent for COVID-19 patients. Hematological and liver diseases were not common and were seen in less than one percent of the total patients in the study (Fig. 1).

Moreover, analysis of the association between the patient comorbidities and mortality yielded interesting results. Approximately, 66% (55/83) of the COVID-19 patients with kidney dysfunction had chronic kidney disease (Stage 3). Kidney disease as a comorbidity for adult COVID-19 patients was found to be detrimental to survival. The mortality rate was almost eight-fold high (19.4%) and statistically significant (p < 0.001), as compared to other COVID-19 infected patients without kidney disease (2.4%) (Table 2). The associated comorbidities such as respiratory, hematological, liver diseases, and immunocompromising disease, and obesity did not significantly impact the survival of adult COVID-19 patients when compared with those without the same morbidities (Table 2).

**Table 2:** Assessment of Association between Patient Comorbidities and Survival for Adult COVID-19 Patients Admitted to the Public Hospitals in Al Ain, the Eastern Region of the United Arab Emirates

Comorbidity	Category	Outcome				P value
		Survived		Deceased		
		n	%	n	%	
Kidney disease	No	2716	97.6	68	2.4	< 0.001
	Yes	83	80.6	20	19.4	
Heart disease	No	2651	97.3	74	2.7	< 0.001
	Yes	148	91.4	14	8.6	
Diabetes Mellitus	No	2069	97.9	45	2.1	< 0.001
	Yes	730	94.4	43	5.6	
Hypertension	No	2100	97.6	52	2.4	< 0.001
	Yes	699	95.1	36	4.9	
Respiratory disease	No	2681	97.1	81	2.9	Not significant
	Yes	118	94.4	7	5.6	
Hematology	No	2781	97	87	3	Not significant
	Yes	18	94.7	1	5.3	
Immunocompromised	No	2727	97	83	3	Not significant
	Yes	72	93.5	5	6.5	
Liver Disease	No	2792	97	87	3	Not significant
	Yes	7	87.5	1	12.5	
Obesity	No	2706	97	83	3	Not significant
	Yes	93	94.9	5	5.1	

To predict the association between critical patient characteristics and comorbidity-related mortality, the 0dds ratio was estimated. Patient age and nationality were chosen for the patient characteristics and, the comorbidities identified included heart disease, hypertension, kidney disease, and patients with diabetes mellitus. Older COVID-19 patients between 51 and 65 years had a significantly lower odds for survival (Crude OR 14.1 95% CI (3.4 – 58.4), p < 0.001) and (Adjusted OR 12.3, 95% CI (2.9–52.4), p < 0.001). Patient age beyond 66 years significantly decreased the odds for survival (Crude OR 36.1, 95% CI (8.5–154.1), P < 0.001) and (Adjusted OR 26.6, 95% CI (5.7–123.8), p < 0.001) (Table 3). Kidney disease, particularly chronic kidney disease, as comorbidity significantly diminished the survival rates for COVID-19 patients (Crude OR 9.6, 95% CI (5.6–16.6), p < 0.001) and (Adjusted OR 5.7, 95% CI (3.0–10.8(, p < 0.001, Table 3), as compared to those without kidney disease. Patient nationality and other comorbidity-related risk factors such as heart disease, hypertension, and diabetes did not significantly alter the odds (adjusted OR) for the survival of COVID-19 patients in our study.

**Table 3:** Odds ratio (crude and adjusted) for predicting the association between critical patient characteristics and comorbidities against mortality for COVID-19 infection

Characteristics	Group	Crude			Adjusted		
		OR	95% CI	P	OR	95% CI	P
Age (years)	18–30	1	–	–	1	–	–
	31–50	3.7	0.9–15.6	Not Significant	3.3	0.8–14.2	Not significant
	51–65	14.1	3.4–58.4	< 0.001	12.3	2.9–52.4	< 0.001
	> 65	36.1	8.5–154.1	< 0.001	26.6	5.7–123.8	< 0.001
Nationality	Asia	1	–	–	1	–	–
	Middle East Arab	1.7	1.1–2.7	< 0.05	0.9	0.5–1.5	Not significant
	Others	2.5	1.3–5.1	< 0.01	2.1	1.0–4.5	Not significant
Heart disease	No	1	–	–	1	–	–
	Yes	3.4	1.9–6.1	< 0.01	1	0.5–2.0	Not significant
Hypertension	No	1	–	–	1	–	–
	Yes	2.1	1.4–3.2	< 0.001	0.8	0.5–1.3	Not significant
Kidney disease	No	1	–	–	1	–	–
	Yes	9.6	5.6–16.6	< 0.001	5.7	3.0–10.8	< 0.001
Diabetes mellitus	No	1	–	–	1	–	–
	Yes	2.7	1.8–4.1	< 0.001	1.3	0.8–2.1	Not significant

Additionally, the majority of patients were not treated with antibiotics (70%, 2154/3072), those severely ill received Amoxicillin Clavulanate, Piperacillin-Tazobactum, Amoxicillin, and Doxycycline. Less than 10% (7.2%, 224/3072) of the patients did not receive any antiviral therapy, others received hydroxyquinoline and Favipiravir as monotherapy, or in combination (data not shown).

## Discussion

Our study describes COVID-19 mortality in a multicultural multi-ethnic population of the United Arab Emirates. Although studies from various countries suggested that mortality was associated with ethnicity [19–23], our study found no significant association between COVID-19 mortality and ethnicity in the UAE. Our results indicate higher age and chronic kidney disease were significantly associated with a higher risk of death (Table 3). Other comorbidity-related risk factors such as heart disease, hypertension, and diabetes were not associated with COVID-19 mortality in UAE.

A population-based cohort study of 17 million adults in England found that minority ethnic groups were at increased risk of contracting COVID-19 disease, related hospitalization, ICU admission, and fatality after ethnic differences in testing were adjusted [19]. Data from the USA, indicate that COVID-19 deaths were more among the African Americans, especially in Chicago, followed by the Hispanics, than the White Americans [20]. Ethnic and regional factors have been associated with increased mortality among hospitalized patients in Brazil [21], and other regions of the world [22, 23]. In Brazil, the regional factors contributed to the increase in the comorbidity burden in regions with low socioeconomic development, while the ethnic factor resulted from the differences in susceptibility to COVID-19 and the access to intensive care [21].

Men were disproportionately affected by COVID-19 as compared to women: less than one-quarter of the COVID-19 patients were women in our study [24]. Higher neutralizing antibodies, smaller lung size, lower chance of immune dysregulation, and the differential expression of the angiotensin-converting enzyme 2-the viral receptor, have been postulated to contribute to the gender difference in COVID-19 infectivity [25]. A large proportion of the UAE population is Asian, especially the South East Asians from Pakistan, India, Bangladesh, Sri Lanka, Philippines, and Nepal [26]. The majority among them are laborers who often live in crowded housing conditions and are at a higher risk for contracting communicable diseases [26] thus explaining the higher COVID-19 infectivity amongst the Asian population (Table 1). Incidentally, lower social status has also been reported to be a risk factor for COVID-19 infection [27, 28]. Increased mortality from COVID-19 disease was seen in the patient cohort that lived in family accommodation (3.9%, p < 0.005, Table 1), as compared to single shared accommodation (1.5%, Table 1). The Middle East Arab population (4.3%, Table 1) living in family housing indicated higher mortality (4.3%, Table 1). The contributing factors may be an increased prevalence of Vitamin D deficiency, and a higher prevalence of cardiovascular risk factors such as insulin resistance and obesity, especially amongst the UAE national population [26]. These observations indicate that although crowded shared housing conditions may contribute towards higher infectivity (Asian population 71.2% vs Middle East Arab 23.3%), the economic factor and underlying health conditions, contribute to higher mortality. In support, a national cohort study of over 7000 subjects has shown that diabetes, impaired fasting glucose, hypercholesterolemia, and hypertension is highly prevalent in young adulthood in the UAE [29]. Comorbidities such as diabetes and hypertension were observed in more than one-quarter of the COVID-19 patients, supporting the earlier observations reported by Guan and Xu that patients with diabetes and hypertension were at a higher risk for severe COVID-19 [30, 31].

Despite the myriad of comorbidities for COVID-19 patients observed in our study such as kidney disease, heart disease, diabetes, and hypertension (Table 2), the multivariate analysis indicated that higher patient age and kidney disease significantly contributed to the COVID-19 mortality rates (Table 3). An eight-fold increase (19.4%, p < 0.001) in mortality rate was observed in COVID-19 patients with kidney disease, as compared to those without kidney disease (2.4%). Odds Ratio estimation further validated that kidney disease as comorbidity significantly lowered the survival rates for COVID-19 patients (Crude OR 9.6, Confidence Interval 5.6–16.6, p < 0.001) and (Adjusted OR 5.7, Confidence Interval 3.0–10.8, p < 0.001, Table 3), as compared to those without kidney disease. Gansevoort & Hilbrands recently demonstrated that COVID-19 patients with kidney disease had higher rates of mortality when compared to the patients with heart disease, diabetes, and hypertension comorbidities combined [32]. Advanced ages of COVID-19 patients between 51 and 65 years, significantly decreased the odds for survival (Adjusted OR 12.3, Confidence Interval 2.9–52.4, p < 0.001), and patient age beyond 66 years significantly enhanced mortality (Adjusted OR 26.6, Confidence Interval 5.7–123.8, p < 0.001) (Table 3). Advancing age may also be associated with various comorbidities and therefore older patient age has been noted as an independent risk factor for COVID-19 mortality [33]. Interestingly, the observations are in accordance with the previously published reports from other COVID-19 disease-affected countries [30–33]. The strengths of the study are that this is the first report describing patient characteristics, socioeconomic status, and clinical outcomes for COVID-19 patients from the UAE, and the data was collected from COVID-19 patients hospitalized at the only two public hospitals in Al Ain that catered to more than 98% of the total positive COVID-19 caseload.

Our study has several limitations: a) the data for the study was retrospectively collected from pre-recorded patent medical records, making it difficult to assess any temporal relationships between the variables; b) the retrospective design of the study enables assessment of association and not causation; c) other confounding factors may have been overlooked, and d) although 85.8% of the COVID-19 patients were smokers (Table 1), the risk did not affect their survival (2.6 Vs 2.4, Smoking Vs Non-Smoking, Table 1), the reasons for which need to be investigated. Data abstraction by multiple abstractors blinded to the study hypothesis and assessment of interrater reliability for the data collected helped to prevent selection bias.

## Conclusion

Our study highlights that COVID-19 patients above 51 years of age had comparatively decreased odds for survival than their younger counterparts. Despite other comorbidity risks, kidney disease contributed to increased mortality by over eight-fold and reduced the odds of survival (Adjusted OR 26.6), compared to those patients without kidney disease. Ethnicity was not associated with increased COVID-19 mortality in the UAE population. Our findings are important in the management of the COVID-19 disease in the region with similar economic, social, cultural, and ethnic backgrounds.

## Data Availability

The datasets generated and/or analyzed during the current study ae not publicly available due to the institutions policy to code and archive data in a central repository of the hospital, but are available from the corresponding author on reasonable request

## Abbreviations

ICH: International Conference for Harmonization
GCP: Good Clinical Practice guidelines
SARS: Severe Acute Respiratory Syndrome
SARS-CoV-2: Severe Acute Respiratory Syndrome Coronavirus 2
RTPCR: Reverse Transcription Polymerase Chain Reaction
UAE: United Arab Emirates
WHO: World Health Organization
